# CLONES : a closed-loop simulation framework for body, muscles and neurons

**DOI:** 10.1186/1471-2202-12-S1-P363

**Published:** 2011-07-18

**Authors:** Thomas Voegtlin

**Affiliations:** 1 INRIA Lorraine, Campus Scientifique, F-54506 Vandoeuvre-les-Nancy, France

## 

The activity of neurons does not only reflect cognitive states, it is also highly constrained by mechanical properties of the body: sensors, muscles, tendons, and their interaction with the environment. Thus, in order to model the activity of neurons, it seems necessary to take a holistic approach, where the behavior of an animal is modeled through the interaction between neurons, muscles and the environment. This involves knowledge in both neurophysiology and biomechanics. It also requires the development of software that can simulate the interaction between neurons and muscles efficiently.

In order to achieve this, we have developed an open-source library called CLONES (Closed Loop Neural Simulation). CLONES is a communication interface between the BRIAN neural simulator [1], and SOFA, a physics engine for biomedical applications [2]. BRIAN and SOFA are both intuitive and high performance simulation environments.

Communication between BRIAN and SOFA is achieved through shared memory and semaphores. A single step of simulation typically takes place on different CPU cores. Once a simulation step has been completed in both simulators, sensory inputs to the neurons and motor outputs to the muscles are updated. SOFA uses an interpreted XML description of a physical scene. The scene can be modified without interrupting the simulation.

CLONES contains a SOFA plugin and a PYTHON module. The Python module provides a function to be called after each step of the simulation. The Sofa plugin provides components for the simulation of muscles, sensors, drag forces. In addition, several demonstration examples are provided with the library. In particular, a neuro-mechanical model of undulatory locomotion in the worm *caenorhabditis elegans*[*3*] is available.
